# A Definitive IMRT-SIB with Concomitant Chemotherapy for Synchronous Locally Advanced Anal Canal Cancer and Prostate Cancer

**DOI:** 10.1155/2018/6101759

**Published:** 2018-06-24

**Authors:** Tubin Slavisa, Raunik Wolfgang

**Affiliations:** Institut für Strahlentherapie/Radioonkologie, Feschnigstraße 11, 9020 Klagenfurt am Wörthersee, Austria

## Abstract

Currently, there are no specific recommendations regarding the management of the synchronous tumours due to the lack of either specific guidelines or individuals' clinical experiences relative to these clinical situations. In the presence of a locally advanced double primary tumour and with the lymph node metastases in addition, from the radiotherapeutical point of view, it must be challenging to manage this complicated situation that requires a more delicate treatment planning, due to higher doses prescribed to greater volumes concomitantly with the chemotherapy. A 68-year-old Caucasian male with a synchronous intermediate-risk prostate adenocarcinoma and locally advanced anal canal carcinoma underwent IMRT-SIB with concomitant chemotherapy at our institute. Two years after the treatment, the restaging CT and MRI scan showed no evidence of the disease and the patient reported no significant gastrointestinal or genitourinary toxicity. Our experience is unique, since it is the first report on using the IMRT-SIB technique simultaneously with chemotherapy in the management of the synchronous prostate and anal canal carcinomas. Therefore, we find it important to provide the current literature with the results from our experience which show good feasibility, efficacy, and tolerability of the definitive concomitant IMRT-SIB-chemotherapy for the synchronous anal canal cancer and prostate cancer.

## 1. Background

Generally speaking, synchronous tumours are very rare clinical entities, and for that reason, the recommendations for their treatment, as well as the epidemiological data, are still lacking in the available papers. However, for some categories, like those found among the synchronous colorectal neoplasms, there are few details that are available in papers that best show how rare these clinical entities are, with the incidence between 0.17% and 0.69%, in the case of 2-3 synchronous lesions [1]. But, for the eventual prostate and anal canal synchronous cancers, the epidemiological data does not exist at all, due to an extremely rare clinical situation. By considering those tumours separately, it has already been well established that the prostate cancer is the most common cancer in Europe for males [2], while the anal canal carcinoma is much less common. The American Cancer Society estimated that, in 2016, approximately 2.6% of the new cases of anal canal, anorectum, or anus cancers, among the digestive system cancers, will be diagnosed [3]. Even though its incidence rate has increased in the recent years, the squamous cell anal carcinoma is still considered a rare malignancy, and in Europe, its annual incidence ranges from 3 per 100,000 (men; Geneva, Switzerland) to less than 1 per 100,000 (both sexes; England and the Netherlands) [4]. Usually, small carcinomas of the anal margin are well treated with local excision, while the concomitant radio-chemotherapy, using 5-fluorouracil and mitomycin C, is a standard first-line treatment for all other cases; salvage surgery is reserved for the local relapses [5]. Based on the tumour stage, a patient's performance status and life expectancy treatment options for the prostate cancer include radical prostatectomy, radiotherapy (external beam radiotherapy and/or brachytherapy), and hormonal therapy [6]. While the existing National Comprehensive Cancer Committee (NCCN) guidelines consider and contain the specific recommendations on the treatment for single tumours, among those mentioned above, there are no recommendations regarding the management of the same tumours when they are synchronous. Considering the fact that, in certain clinical situations, the definitive radiotherapy and/or radio-chemotherapy for those tumours could induce significant toxicity, even when treated individually, we find it important to report on managing an even more complicated situation than this, with synchronous locally advanced tumours that require a more delicate treatment planning, due to higher doses prescribed to greater volumes concomitantly with chemotherapy.

Therefore, we thought that reporting the results of our experience shall be interesting, due to the lack of either specific guidelines or individuals' clinical experiences relative to these kinds of clinical situations, and it can provide the currently available papers with the results on feasibility, efficacy, and tolerability of the definitive concomitant IMRT-SIB-chemotherapy for the synchronous locally advanced anal canal cancer and intermediate-risk prostate cancer.

## 2. Case Description

The current disease story of our 68-year-old Caucasian male started two years ago, when this patient observed a hard lesion in the anal canal, which, in the first months since he observed it, was neither officially diagnosed nor treated. Ten months after that, he underwent cholecystectomy due to cholelithiasis, and the perioperative diagnostic examination revealed some suspicious lesions in the anal canal and some enlarged pelvic lymph nodes. His comorbidities included only gout, and his Karnofsky performance status was 90. He has never been treated with radiotherapy, and he had a second-degree family history for malignancies. At that time, a digital rectal examination identified mild prostatomegaly, with a palpable suspicious nodule in the left prostate lobe, without any signs of the extracapsular extension. Also, a large ulcerated mass was palpable at the dorsal anal canal wall, without lymphadenopathies in the inguinal region. Subsequent colonoscopy confirmed the presence of a suspicious lesion on the posterior anal canal, extending above up to the anorectic junction. A magnetic resonance imaging (MRI) for the regional staging confirmed an infiltrative lesion of the anal canal with a maximum diameter of 3.2 cm, with a high-grade suspicion of sphincter infiltration, and two enlarged lymphadenopathies in the mesorectum and in the right internal iliac region, respectively, both with a maximal diameter of 2 cm, which is suspicious of tumour metastases. Further, an area with a maximal diameter of 2 cm within the left prostate basis having a restricted diffusion with a lower apparent diffusion coefficient (ADC) than that in the surrounding healthy prostate tissue, which appeared hypointense on ADC maps but hyperintense on the diffusion-weighted maps, was also described. Afterwards, biopsies confirmed a poorly differentiated squamous cell carcinoma of the anal canal, as well as a moderately differentiated prostate adenocarcinoma, with a Gleason score of 7 (3 + 4) in both lobes. He resulted human immunodeficiency virus- (HIV-) negative, and his carcinoembryonic antigen (CEA) and prostate-specific antigen (PSA) were 2.09 U/L (reference range = 0–5.0 U/L) and 0.74 ng/mL (reference range = 0–4.0 ng/mL), respectively. Additional staging with the whole-body computed tomography (CT) scans excluded the presence of any distant metastases. Therefore, our patient had a synchronous intermediate-risk stage IIB cT2cN0M0 prostate adenocarcinoma and stage IIIB cT2N2M0 anal canal squamous cell carcinoma.

A multidisciplinary team which included surgical, medical, and radiation oncologists evaluated this case and passed a decision to submit the patient to the definitive concurrent radio-chemotherapy.

Regarding this clinical entity, the current papers are “blind,” and at this moment, they can provide us only with one case report on early-stage I metachronous small anal canal squamous cell carcinoma (cT1N0) and intermediate-risk prostate cancer (cT1cN0) that was published in 2011 [7]. Due to the relative lack of experience in the intensity-modulated radiation therapy (IMRT) at the time, which should be a more appropriate radiotherapy technique for managing this kind of conditions, the authors planned a conventional 3D conformal radiotherapy by using a wide anterior-posterior/posterior-anterior field arrangement with concurrent mitomycin C (12 mg/m^2^ i.v. bolus on day 1) and 5-fluorouracil (1000 mg/m^2^ on days 1–4 (week 1) and 29–32 (week 5) by continuous 24 h i.v. infusion). Due to the use of this technique, the prescribed doses for the anal canal carcinoma and the prostate carcinoma were 50.4 Gy and 73.8 Gy, respectively. That treatment has been well tolerated, without any significant gastrointestinal or genitourinary toxicity, except for one-week treatment break, due to the moist desquamation in the bilateral inguinal and intergluteal areas. The last restaging eighteen months later showed no evidence of the disease or of the cancer whatsoever.

## 3. Treatment Planning

**Figure 1:**
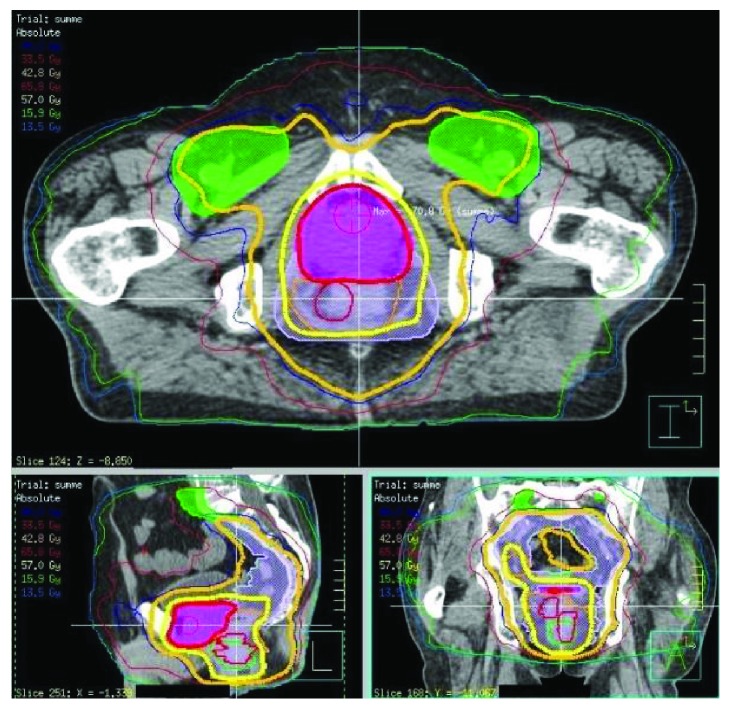
Dose distribution in the sum plan at the level of both primary tumours.

**Figure 2:**
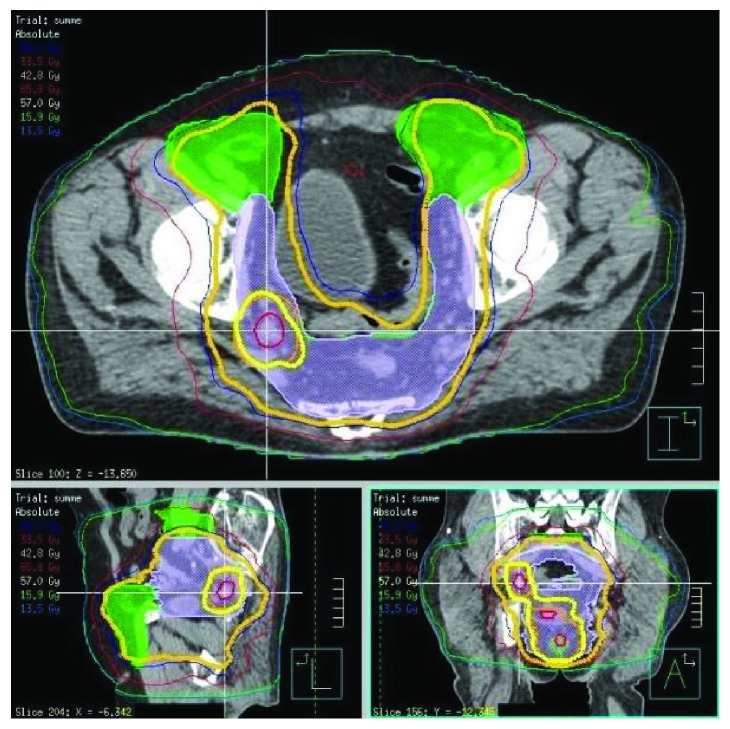
Dose distribution in the sum plan at the level of the right internal iliac lymph node metastasis.

The presence of the two synchronous cancers, as in our abovementioned case, which should be treated at the same time, is a difficult circumstance in itself, and our case was even further complicated with the four nearby volumes that should have been covered with a high-dose radiotherapy, due to the presence of the locally advanced cT2N2 anal canal carcinoma. Therefore, our choice was the IMRT-SIB technique targeted at improving the therapeutic ratio. The simulation of the treatment was performed in the supine position, with a comfortably full bladder, after which the planned CT (performed with 2 mm slice thickness) was then fused with the diagnostic MRI for the subsequent treatment planning. FeetSTEP and KneeSTEP were used in combination, to achieve the maximum accuracy in positioning and repositioning the hip and lower limbs. In order to ensure the best possible localization and contouring of the small bowel, the patient got three deciliters of the oral contrast 60 minutes prior to the simulation. The anal marker was used to mark the anal verge. Also, the patient was instructed on what diet to maintain during the treatment to ensure the regular bowel function. The IMRT-SIB plan was designed by using Pinnacle^3^ Treatment Planning (Philips) (Figures 1 and 2).

Elective clinical target volume (CTV) included the anal canal with the perineum, the entire mesorectum (perirectal nodal region) to the pelvic floor, the inguinal and pelvic nodal regions (internal iliac, external iliac, and presacral). For the contouring of the elective nodal CTV, the 8 mm margin in soft tissue (excluding uninvolved bone and muscle but including small lymph nodes) around the iliac blood vessels has been added. The inferior extent of the inguinal nodal region was 2 cm caudal to the saphenous/femoral junction. CTV included 2 cm of the apparently normal perianal skin around the anal verge, 1 cm of the posterior bladder to account for day-to-day variation in the bladder position, and just a 5 mm beyond the levator muscles in the lower pelvis. The posterior and lateral margins of CTV extended to pelvic sidewall musculature or, where absent, bone. The superior extent of CTV was where the common iliac vessels bifurcate into external/internal ilia (approximate boney landmark: sacral promontory) including the rectosigmoid junction. At midline, CTV extended 1 cm anterior to the sacrum, to cover properly the presacral nodal region. Boost CTV was created by adding a 5 mm margin to the involved pelvic lymph nodes and a 2 cm margin on the gross tumour within the anal canal. An additional margin of 8 mm was added to CTV to create the planning target volume (PTV). For a definition of the target volumes, the anorectal contouring atlas published by RTOG [8] was used. CTV prostate included a whole prostate gland together with a neurovascular bundle and 1 cm of the proximal seminal vesicles to which an additional 7 mm margin was then added to create prostate PTV.

As regards the tumour extension, the four different PTV dose volumes were defined:

1. PTV1: the uninvolved inguinal lymph nodes (cN0) have been irradiated with a single dose of 1.8 Gy up to 36 Gy.
2. PTV2: the uninvolved pelvic lymph nodes (cN0) have been irradiated with a single dose of 1.8 Gy up to 45 Gy.
3. PTV3: the involved pelvic lymph nodes (cN2) and primary tumour of the anal canal have been irradiated with a single dose of 1.8 Gy up to 59.4 Gy.
4. PTV4: the prostate has been irradiated with a single dose of 2.1 Gy up to 69.3 Gy.

The dose that was prescribed to the prostate (PTV4) was slightly hypofractionated and has biologically corresponded to the 72 Gy equivalent dose in 2 Gy fractions (EQD2) considering the *α*/*β* ratio of 1.2/1.5 Gy for the prostate cancer. Before being delivered with 11 fields by 15 MV LINAC, the radiotherapy plan has been submitted for the quality assurance, to be approved for the delivery accuracy. To ensure good tolerability of the treatment that was performed, the constraints published by RTOG have been met [9]. The toxicity was assessed with the CTCAE score. The patient was regularly followed up weekly during the treatment and then every 4 months after the completion of treatment.

## 4. Results

The patient has tolerated chemoradiotherapy very well presenting only with an acute toxicity grade 2 erythema in the perianal region, which did not require the suspension of his treatment, and grade 1 anemia. During the follow-up, few months after his treatment, he reported very mild late radiation proctitis and fecal incontinence which regressed spontaneously without requiring any medical treatment. At his last follow-up two years after the treatment, the restaging CT and MRI scan showed no evidence of the disease and he reported no gastrointestinal or genitourinary toxicity.

## 5. Conclusions

As already mentioned, the current papers present only one case report on the metachronous early-stage anal canal cancer and prostate cancer, so this is the first report on using the IMRT-SIB technique simultaneously with chemotherapy in the management of the synchronous prostate and anal canal carcinomas. By using this treatment option and by achieving a complete remission among all four evident tumour sites, with the acceptable low toxicity profile, we conclude that this therapeutic option is feasible, effective, and well tolerable.
